# GLP-1 receptor agonists may enhance the effects of desmopressin in individuals with AVP deficiency: a case series and proposed mechanism

**DOI:** 10.1007/s11102-024-01451-7

**Published:** 2024-09-06

**Authors:** Afif Nakhleh, Naim Shehadeh, Bshara Mansour

**Affiliations:** 1 Maccabi Healthcare Services, Haifa, Israel; 2 Institute of Endocrinology, Diabetes and Metabolism, Rambam Health Care Campus, Haifa, Israel; 3 Azrieli Faculty of Medicine, Bar-Ilan University, Safed, Israel; 4 Diabetes and Endocrinology Clinic, Maccabi Healthcare Services, 54 Simcha Golan St., Haifa, Israel

**Keywords:** AVP deficiency, Desmopressin, GLP-1 RAs, Natriuresis

## Abstract

**Background:**

Glucagon-like peptide-1 receptor agonists (GLP-1 RAs) have diverse effects on sodium and water homeostasis. They decrease thirst perception, potentially inhibit arginine vasopressin (AVP) production, and induce natriuresis. We present three cases of AVP deficiency (AVP-D) where GLP-1 RA initiation led to desmopressin dose reduction.

**Cases:**

Three patients with AVP-D on stable desmopressin therapy started GLP-1 RAs for type 2 diabetes mellitus or obesity. Following weight loss and decreased thirst, all patients reduced their desmopressin dose while maintaining normal thirst and urine output.

**Discussion:**

GLP-1 RAs influence sodium and water homeostasis through various mechanisms. In individuals with intact AVP systems, GLP-1 RAs may directly suppress AVP production and induce natriuresis in the kidney leading to increased water excretion. In AVP-D, with exogenous desmopressin replacing endogenous AVP, the osmotic permeability of collecting ducts is primarily influenced by desmopressin dose. Thus, increased distal fluid delivery may allow for lower desmopressin doses to maintain water balance.

**Conclusion:**

Our findings indicate a potential interaction between GLP-1 RAs and desmopressin in AVP-D. Clinicians should reassess desmopressin dosage upon initiating GLP-1 RA therapy.

## Introduction

Glucagon-like peptide-1 (GLP-1) plays a multifaceted role in maintaining sodium and water homeostasis in humans. In the brain, GLP-1 plays a direct role in modulating thirst perception and leads to decreased water intake [1]. It is also thought to directly inhibit arginine vasopressin (AVP) production [2]. In the gut, GLP-1 reduces sodium absorption [1]. In the kidney, GLP-1 has a natriuretic effect and can potentially enhance renal hemodynamics [3].

The viability of GLP-1 receptor agonists (GLP-1 RAs) as a potential treatment for primary polydipsia has come to attention recently. Winzeler et al. presented evidence that suggests the potential of GLP-1 RAs as a viable therapeutic option for primary polydipsia. Patients with primary polydipsia who were administered dulaglutide, a GLP-1 RA, showed a significant reduction in fluid intake and thirst perception as compared to those who were given a placebo [4].

AVP deficiency (AVP-D), also known as central diabetes insipidus, is another cause for polyuria-polydipsia syndrome. This condition is characterized by hypotonic polyuria (50 ml/kg body weight per 24 h) and polydipsia (> 3 L per day). Desmopressin is the primary treatment for AVP-D. It targets AVP receptor 2 and improves excessive urination and thirst [5].

In this report, we present three patients diagnosed with AVP-D who required a reduction in their desmopressin dosage following initiation of GLP-1 RA. We propose a hypothesis to explain why desmopressin's antidiuretic effect may be increased when combined with GLP-1 RA.

## Cases presentation

### Patient 1

A 70-year-old man with a history of AVP-D following resection of a third ventricle colloid cyst 24 years ago. He was maintained on a stable daily dose of desmopressin (400 mcg) for the past 5 years, with a weekly intentional omission of 200 mcg. His condition was well-controlled without symptoms of polydipsia or polyuria (Table 1). The anterior pituitary function was normal. The patient presented to our clinic for the management of a recently diagnosed type 2 diabetes mellitus (T2DM). Comorbid conditions included obesity (BMI = 30.3 kg/m^2^), hypertension managed with enalapril (20 mg/day) and lercanidipine (10 mg/day), hyperlipidemia on atorvastatin (40 mg/day) and depression treated with escitalopram (10 mg/day). He was started on semaglutide, and the dose was gradually increased to 1 mg weekly. Following 4 months of semaglutide treatment, he reported a 6 kg weight loss (98 to 92 kg) and a noticeable reduction in thirst and desire to drink water, but no significant change in urinary frequency. Laboratory evaluation showed mild hyponatremia (Table 1). His desmopressin dose was gradually tapered to 200 mcg daily (decreased by 200 mcg/day). Three months later, the patient reported a return to normal thirst levels, and his 24-h urine volume was 2400 ml. Further laboratory results are provided in Table 1.

**Table 1:** Patient characteristics

	Parameter	Before GLP-1 RA initiation	3–4 months after GLP-1 RA initiation	3 months after desmopressin dose reduction
Patient 1 A 70-year-old man	eGFR^a^ (ml/min/1.73m^2^)	95	99	97
	Serum sodium (mmol/l)	138	132	138
	Blood osmolality (mOSM/kg/H2O)	285	268	282
	Urine osmolality (mOSM/kg/H_2_O)	732	817	701
	HbA1c (%)	6.5	5.5	5.8
	Weight (kg)	98	92	91
	BMI (kg/m^2^)	30.3	28.4	28.1
	24-h urine volume (ml)	2600	N/A	2400
Patient 2 A 49-year-old woman	eGFR^a^ (ml/min/1.73m^2^)	109	105	103
	Na (mmol/l)	138	139	141
	Blood osmolality (mOSM/kg/H2O)	282	283	284
	Urine osmolality (mOSM/kg/H2O)	730	766	652
	HbA1c (%)	5.7	N/A	N/A
	Weight (kg)	99	92	87
	BMI (kg/m^2^)	34.3	31.8	30.1
	24-h urine volume (ml)	2200	N/A	2300
Patient 3 A 67-year-old woman	eGFR^a^ (ml/min/1.73m^2^)	80	77	77
	Na (mmol/l)	142	140	138
	Blood osmolality (mOSM/kg/H2O)	295	289	283
	Urine osmolality (mOSM/kg/H2O)	763	723	753
	HbA1c (%)	7.2	6	N/A
	Weight (kg)	87	82	82
	BMI (kg/m^2^)	33.5	31.6	31.6
	24-h urine volume (ml)	2050	N/A	2100

*24-h* 24-h, *BMI* body mass index, *eGFR* estimated glomerular filtration rate, *HbA1c* glycated hemoglobin, *N/A* not available

^a^Estimated glomerular filtration rate was calculated using the 2009 chronic kidney disease epidemiology collaboration (CKD-EPI) equation

### Patient 2

A 49-year-old woman with a history of AVP-D following resection of a non-secreting pituitary macroadenoma 4 years ago, currently controlled with desmopressin 250 mcg/day. The patient followed a once weekly desmopressin omitting strategy. Her medical history includes secondary hypothyroidism well-controlled with levothyroxine (100 mcg/day), depression treated with venlafaxine (37.5 mg/day), psoriatic arthritis on methotrexate (10mg weekly) and obesity (BMI = 34.3 kg/m^2^). She underwent single anastomosis gastric bypass 5 years ago.

Liraglutide was initiated for weight management, with a gradual increase in dose over 4 weeks to 3mg/day. After four months of liraglutide therapy, she experienced a weight reduction from 99 to 92 kg. The patient reported a modest decrease in thirst and desire to drink water, with no alterations in urinary frequency. Laboratory assessments were performed (Table 1), and the desmopressin dose was titrated down to 150 mcg/day (decreased by 100 mcg/day). Three months later, she reported returning to her previous thirst and drinking habits, and her 24-h urine volume was 2300 ml. Additional laboratory findings are presented in Table 1.

### Patient 3

A 67-year-old woman, with a 14-year history of AVP-D following surgical removal of a pituitary stalk granular cell tumor, was maintained on desmopressin (200 mcg/day) with a weekly omission strategy. She has secondary hypoadrenalism, treated with prednisolone (5 mg/day), secondary hypothyroidism well-controlled on levothyroxine (100 mcg/day), hypertension controlled with valsartan (160 mg/day) and amlodipine (5 mg/day), hyperlipidemia managed with atorvastatin (20 mg/day) and a newly diagnosed T2DM. For the management of her diabetes, the patient was started on semaglutide, with the dose gradually increased to 1 mg weekly over two months. After three months of semaglutide treatment, the patient reported weight loss (from 87 to 82 kg) and a slight decrease in thirst and water intake, but no change in urinary frequency (Table 1). Consequently, her desmopressin dose was reduced to 100 mcg/day (decreased by 100 mcg/day). Three months later, the patient reported a return to normal thirst, and her 24-h urine volume was 2100 ml. Detailed laboratory results are in Table 1.

## Discussion

We present three patients with a history of AVP-D and stable desmopressin treatment who initiated GLP-1 RA therapy for type 2 diabetes mellitus or obesity. Following weight loss and a self-reported decrease in thirst, all three patients were able to reduce their desmopressin dosage while maintaining normal thirst and urine output. None of the patients were taking diuretics, lithium, or following a low-sodium diet. Although all patients experienced weight loss after starting GLP-1 RAs, this 6–7% reduction in weight is insufficient to explain the 40–50% decrease in desmopressin dosage. These cases suggest a potential impact of GLP-1 RAs on desmopressin requirements in AVP-D. Herein, we discuss the possible interactions between these medications and propose a hypothesis to explain the enhanced antidiuretic effect of desmopressin when combined with GLP-1 RA.

GLP-1 plays a complex role in regulating sodium and water homeostasis. It acts on the brain to decrease thirst and potentially suppress AVP production. GLP-1 has been shown to significantly reduce water intake by 36% in healthy subjects after a salty meal, without affecting their blood sodium levels [1]. This effect is thought to be mediated by a direct influence on drinking behavior, as supported by evidence in rats demonstrating reduced fluid intake with GLP-1 RAs independent of food intake [6]. Interestingly, a recent study utilizing Brattleboro rats, a model of hereditary hypothalamic diabetes insipidus, revealed an augmented response to centrally administered GLP-1 RAs, leading to a greater reduction in fluid intake compared to wildtype rats [7]. However, both wildtype and Brattleboro rats exhibited similar reductions in food intake following GLP-1 RA treatment. This suggests that Brattleboro rats may have a specific dysfunction in the GLP-1 pathway that regulates water intake [7].

In rats, GLP-1 receptor signaling in hypothalamic neurons can directly inhibit the production of AVP [2]. Furthermore, GLP-1 could decrease water consumption by reducing sodium absorption in the gut via inhibition of the intestinal sodium-hydrogen exchanger-3 (NHE3) [1].

The GLP-1 receptor is expressed in various renal locations, including preglomerular vascular smooth muscle cells and juxtaglomerular cells [8]. Multiple studies on experimental models, healthy volunteers, overweight individuals, and diabetic patients have shown that GLP-1 RAs stimulate natriuresis and diuresis [9–12]. This effect is likely mediated by NHE3 inhibition in the proximal renal tubule. When GLP-1 binds to its receptor, it activates protein kinase A (PKA). This activation leads to the phosphorylation of NHE3, which ultimately results in the inhibition of sodium reabsorption in the proximal tubule leading to less fluid reabsorption at this site and increased distal delivery [12, 13].

In the kidney, AVP binds to vasopressin V2 receptors, regulating urine concentration by increasing sodium reabsorption in the thick ascending limb of the loop of Henle and enhancing aquaporin 2 (AQP-2) expression in the apical membrane of collecting duct principal cells, thereby increasing their osmotic permeability [14].

**Fig. 1:**
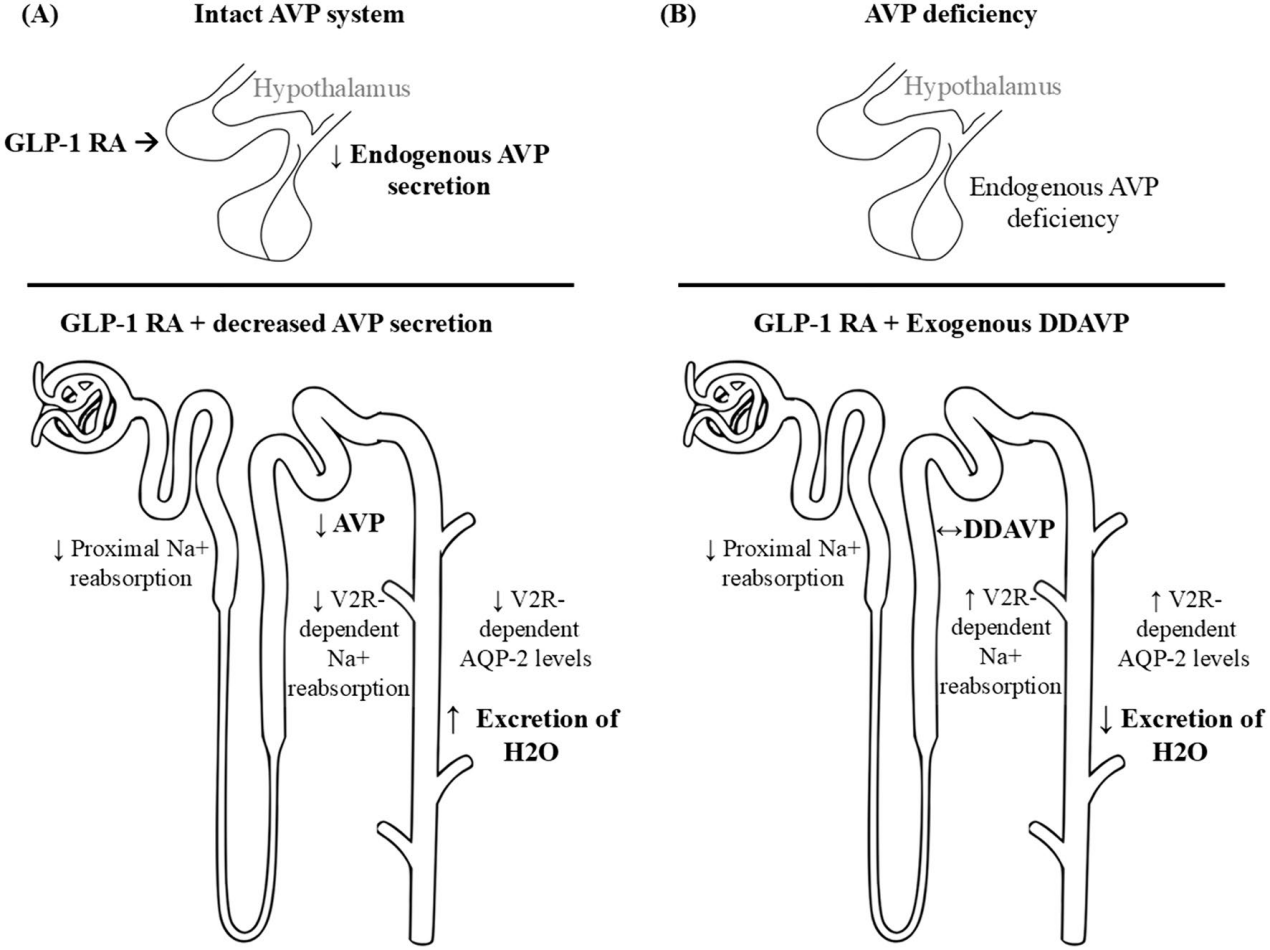
Proposed mechanism of GLP-1 RA impact on sodium and water homeostasis in the kidney. **A** In individuals with normal AVP production, administration of GLP-1 RAs decreases AVP secretion and induces natriuresis. Reduced endogenous AVP levels result in decreased V2 receptor-mediated sodium reabsorption in the distal nephron. Additionally, reduced endogenous AVP levels decrease the osmotic permeability of collecting ducts, leading to increased water excretion. **B** GLP-1 RAs promote natriuresis and increased fluid delivery to the distal nephron. In patients with AVP-D receiving desmopressin replacement, V2 receptor-mediated sodium reabsorption and collecting duct water permeability are primarily regulated by exogenous desmopressin dosage, not endogenous AVP levels. This may account for the observed amplification of desmopressin effects

In individuals with intact AVP system, GLP-1 RA treatment is thought to reduce AVP secretion and induce natriuresis, increasing fluid delivery to the distal nephron and collecting ducts. The GLP-1 RA-induced decrease in AVP should reduce the osmotic permeability of collecting duct, allowing excretion of sodium-free water and restoring osmotic homeostasis (Fig. 1A).

We propose that GLP-1 RAs will also induce natriuresis and increased distal fluid delivery in patients with AVP-D. However, as these individuals receive exogenous desmopressin to replace AVP, the osmotic permeability of the collecting ducts is primarily influenced by desmopressin dosage, not endogenous AVP levels. This may account for the observed amplification of desmopressin effects and subsequent reduction in desmopressin dosages in our cases (Fig. 1B). In other words, the increased distal fluid delivery caused by GLP-1 RAs may allow for lower desmopressin doses to maintain water balance.

This hypothesis is supported by observations of increased hyponatremia risk when desmopressin is combined with other medications that increase distal fluid delivery in the nephron, such as thiazides [15].

GLP-1 RAs may interact directly with the renin–angiotensin–aldosterone system (RAAS) by inhibiting angiotensin II formation, although their effect on renin release remains unclear. Two potential mechanisms have been proposed: indirect inhibition of renin release through tubuloglomerular feedback activation secondary to natriuresis (induced by NHE3 in the proximal tubule), and direct inhibition of angiotensin II production in tissues [16]. Nevertheless, this interaction does not appear to undermine our hypothesis.

In the case of patient 3, who is treated with valsartan, an angiotensin II type 1 receptor (AT1R) antagonist, an additional interaction may exist. AT1R antagonists inhibit sodium reabsorption in the proximal tubule leading to natriuresis via activation of unblocked angiotensin II type 2 receptors by angiotensin III [17]. This could theoretically attenuate the natriuretic effect of GLP-1 RAs. Despite this, patient 3 was able to decrease her desmopressin dosage by 50% after initiating GLP-1 RA.

In the kidneys, the angiotensin-converting enzyme (ACE) maintains a balance between the vasodilatory and natriuretic actions of bradykinin and the vasoconstrictive and salt-retentive effects of angiotensin II. By disrupting this balance, ACE inhibitors promote natriuresis [18]. This could theoretically weaken the natriuretic effect of GLP-1 RAs in patients taking ACE inhibitors. However, patient 1, who was on enalapril, still achieved a 50% reduction in desmopressin dose after starting GLP-1 RA treatment.

While desmopressin can increase hyponatremia risk in older adults [19], this does not explain the 50% dosage decrease in patients 1 and 3, who had stable doses and normal serum sodium levels before GLP-1 RA initiation.

Another factor to consider in patients 2 and 3 is their secondary hypothyroidism. If chronically uncontrolled, it may be associated with a decreased capacity for free water excretion and hyponatremia. This is due to elevated AVP levels, mainly attributed to the hypothyroidism-induced decrease in cardiac output [20]. This interaction seems less relevant in patients 2 and 3, who have AVP-D and were well-controlled with levothyroxine.

An additional factor to consider in patient 3 is secondary hypoadrenalism. Glucocorticoid deficiency can lead to impaired renal free water clearance, causing water retention and dilutional hyponatremia [21]. Additionally, cortisol deficiency stimulates the hypothalamus to increase production of corticotropin-releasing hormone (CRH), which in turn promotes the secretion of AVP [21]. However, our patient has AVP-D and her hypoadrenalism was controlled with prednisolone. Therefore, these interactions are unlikely to be relevant in this case.

This report is limited by its small sample size, observational design, reliance on self-reported data, and lack of specific measurements such as urinary sodium, which precluded assessment of fractional excretion of sodium.

Nonetheless, existing evidence supports our hypothesis and provides a plausible explanation for the observed decrease in desmopressin requirements in patients with AVP-D treated with GLP-1 RAs. However, further research is needed to confirm this hypothesis and elucidate the underlying mechanisms.

## Data Availability

The raw data supporting the conclusions of this article will be made available by the authors without undue reservation.

## Abbreviations

AQP-2: Aquaporin 2
AVP: Arginine vasopressin
DDAVP: Desmopressin
GLP-1 RA: Glucagon-like peptide-1 receptor agonist
H2O: Water
Na +: Sodium ions
V2R: Vasopressin V2 receptors
